# Correlation between “Nutcracker phenomenon” and venous hypertension of the lower extremities: Three case reports

**DOI:** 10.1016/j.jvscit.2022.10.016

**Published:** 2022-10-30

**Authors:** Yuji Hoshino, Hiroyoshi Yokoi

**Affiliations:** a Vascular Surgery, Fukuoka Sanno Hospital, Fukuoka, Japan; b Cardiovascular Medicine, Fukuoka Sanno Hospital, Fukuoka, Japan

**Keywords:** Deep venous insufficiency, Gonadal vein reflux, Nutcracker phenomenon, Pelvic venous disorders, Renal vein compression, Renal vein stenting

## Abstract

We report three cases of left renal vein (LRV) compression resulting in deep venous insufficiency of the lower extremities, among which two patients showed clinical symptoms in both lower extremities. All patients were treated successfully with LRV stenting. The pathophysiology suggested that the secondary gonadal vein reflux owing to LRV compression drained into the iliac veins, resulting in a volume overload and venous hypertension in the iliocaval region. Computed tomography angiography was useful in diagnosing this condition, allowing the evaluation of LRV compression, identification of early opacification of the gonadal vein reflux, and exclusion of other pathologies resulting in deep venous insufficiency of the lower extremities.

The most common etiologies of deep venous insufficiency of the lower extremities (DVI-LE) are post-thrombotic syndrome (PTS) and nonthrombotic iliac vein lesions (NIVL). Left renal vein (LRV) compression between the superior mesenteric artery and aorta, the Nutcracker phenomenon, may cause hematuria, flank pain, pelvic congestion symptoms, and varicosities, and is not considered a cause of DVI-LE. Venous insufficiency in the pelvic region and lower extremities caused by LRV compression and/or left gonadal vein (LGV) reflux has been reported previously.^1^, ^2^, ^3^, ^4^, ^5^, ^6^, ^7^, ^8^, ^9^, ^10^ Meissner et al^1^^,^^2^ categorized them using a novel concept of pelvic venous disorders (PeVD), reporting a variety of clinical presentations, and introduced the symptoms-varices-pathophysiology classification. However, DVI-LE has not yet been described as a clinical manifestation of PeVD. Herein, we report three cases of LRV compression-induced LGV reflux resulting in DVI-LE, which were successfully treated with LRV stenting. The patients consented to publication of this case report.

## Case reports

### Case 1

**Fig 1:**
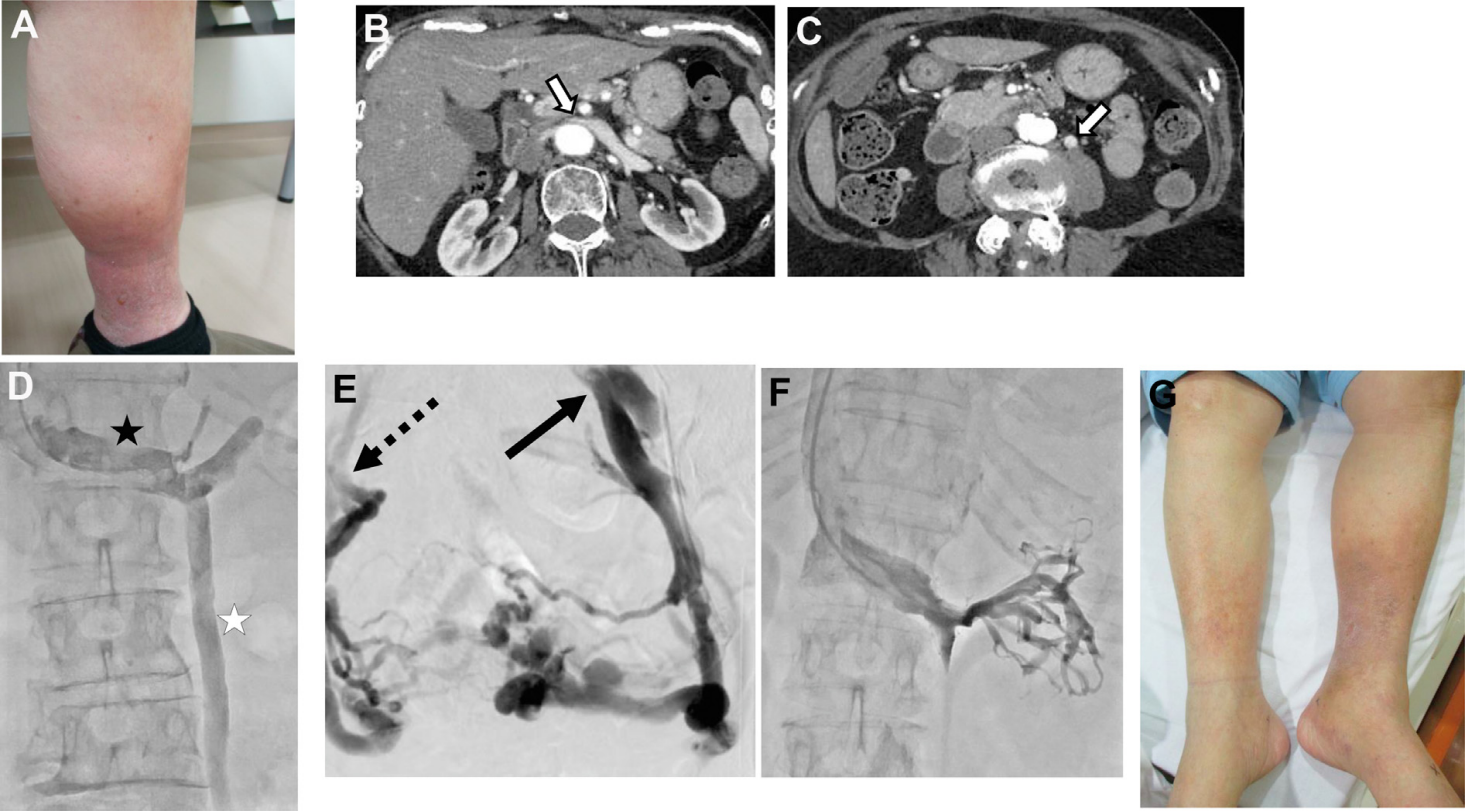
Case 1. **(A)** Preoperative photograph of the patient demonstrating swelling, hyperpigmentation, and serous exudate on her left lower extremity. **(B)** Computed tomography angiography (CTA) showing the left renal vein (LRV) compression between the superior mesenteric artery and the aorta (white arrow). **(C)** Early opacification of the left gonadal vein (LGV) (white arrow). **(D)** Venogram shows compression of the LRV (black star) and the collateral pathway (LGV reflux) (white star). **(E)** Selective left gonadal venography demonstrates LGV reflux drained into the left common iliac vein (CIV) (black arrow) via the pelvic venous plexus (some into the right internal iliac vein; dashed black arrow). **(F)** After LRV stenting, the collateral pathway was reduced. **(G)** The symptoms in her left lower extremity improved postintervention day 1.

A 75-year-old woman presented to our hospital with left lower extremity swelling, hyperpigmentation, and serous exudate (Fig 1, A). No obstruction or reflux was observed in the superficial or deep veins of the lower extremities on duplex ultrasound (DUS) examination. Computed tomography angiography (CTA) revealed LRV compression and LGV early opacification (Fig 1, B and C). A venogram showed LRV compression-induced LGV reflux (Fig 1, D) draining into the left common iliac vein (CIV) via the pelvic venous plexus (or into the right internal iliac vein) (Fig 1, E).

LRV stenting (SMART 14 × 40 mm; Cordis Corporation, Miami, FA) was performed using the jugular approach,^3^^,^^4^ which decreased the LGV reflux (Fig 1, F). The swelling and serous exudate improved on postintervention day 1 (Fig 1, G). The patient was on antiplatelet therapy for 6 months and had good long-term stent patency with no symptom recurrence at the final follow-up 42 months after intervention.

### Case 2

**Fig 2:**
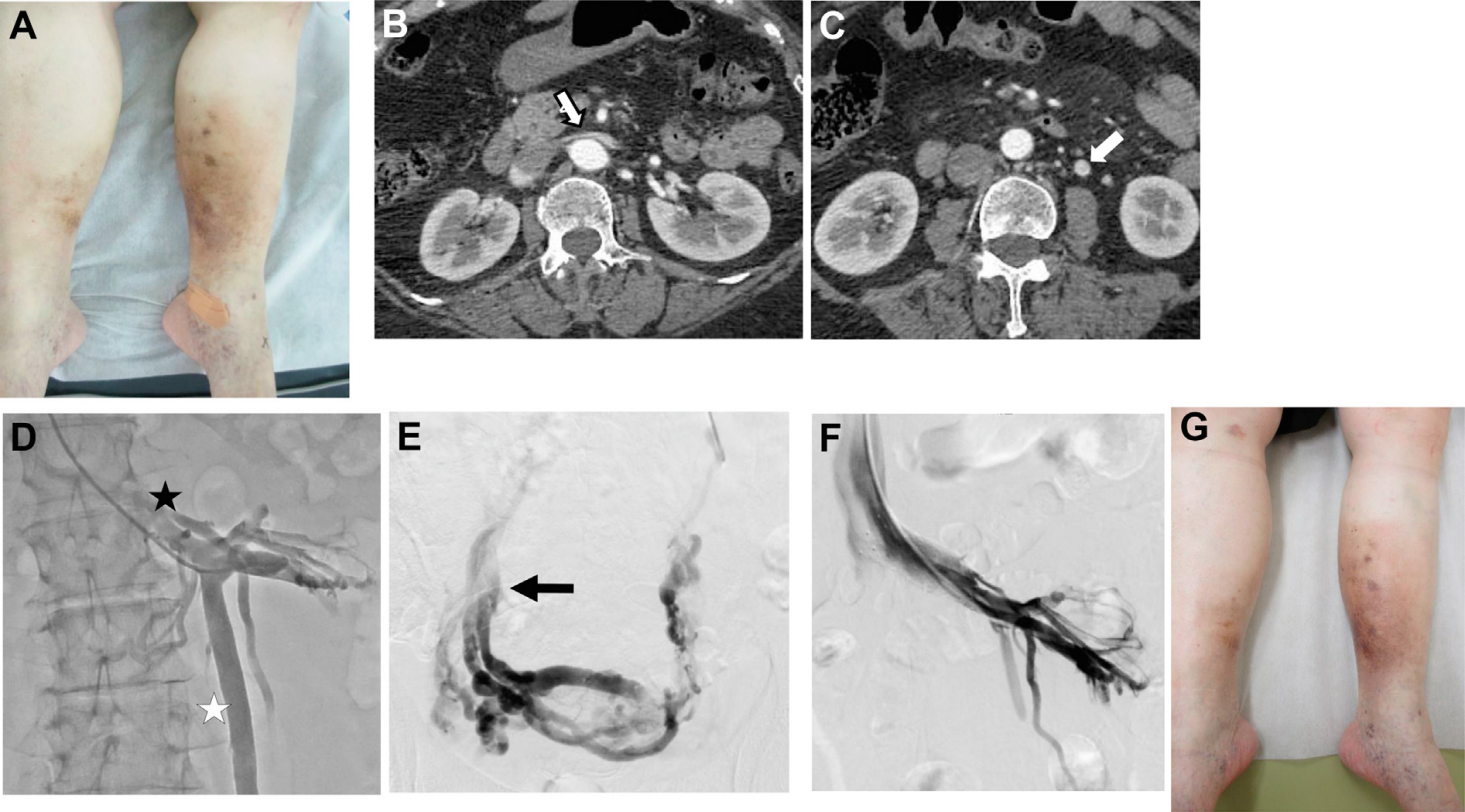
Case 2. **(A)** Preoperative photograph of the patient showing swelling, dermatitis, and hyperpigmentation of both lower extremities, and a bleeding ulcer on her left leg. **(B)** Computed tomography angiography (CTA) showing the left renal vein (LRV) compression between the superior mesenteric artery and the aorta (white arrow). **(C)** Early opacification of the left gonadal vein (LGV) (white arrow). **(D)** Venogram shows compression of the LRV (black star) and the collateral pathway (LGV reflux) (white star). **(E)** Selective left gonadal venography demonstrates reflux in the LGV associated with pelvic varices draining into the right common iliac vein (CIV) (black arrow). **(F)** After LRV stenting, the collateral pathway was reduced. **(G)** One-month postoperative photograph showing improvement in lower extremity symptoms.

A 66-year-old woman with a history of multiple superficial venous surgeries presented to our hospital with swelling, dermatitis, and hyperpigmentation of both lower extremities, and a bleeding ulcer on her left leg (Fig 2, A). DUS examination showed no superficial venous insufficiency, but axial reflux in the bilateral femoral veins (FVs). CTA demonstrated LRV compression and LGV early opacification (Fig 2, B and C). A venogram revealed LRV compression-induced LGV reflux (Fig 2, D) draining into the right CIV via the pelvic plexus (Fig 2, E). We performed LRV stenting (SMART 14 × 60 mm, Cordis Corporation) using the jugular approach.^3^^,^^4^ After stenting, the collateral pathways disappeared (Fig 2, F), and the swelling and dermatitis improved gradually (Fig 2, G). The patient was on antiplatelet therapy for 5 months and had good long-term stent patency with no recurrence of bleeding at the final follow-up 37 months after intervention.

### Case 3

**Fig 3:**
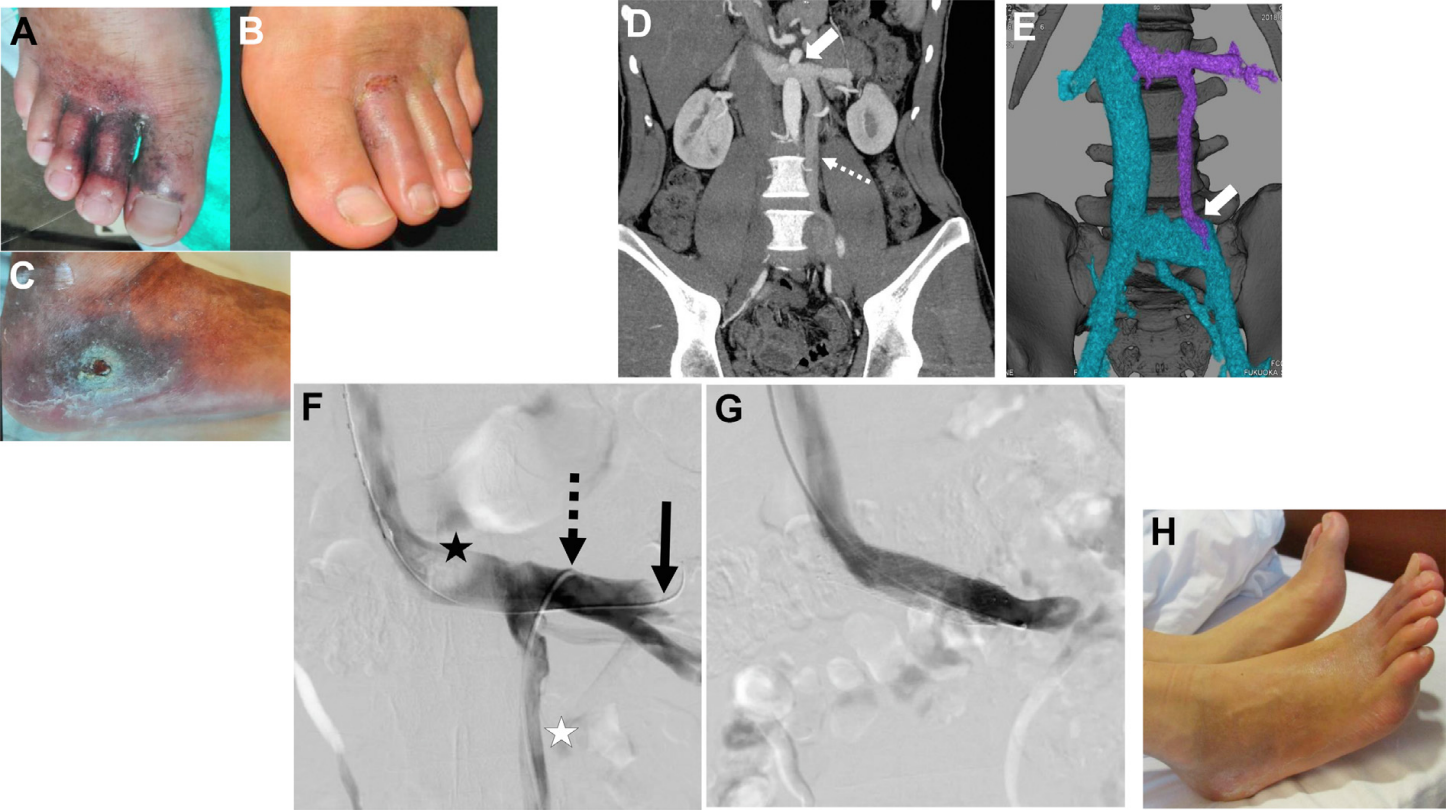
Case 3. **(A-C)** Preoperative photograph of the patient demonstrating ulcers over the lateral ankle and forefoot bilaterally. **(D, E)** Computed tomography angiography (CTA) demonstrates compression of the left renal vein (LRV) (**D**, white arrow), and dilatation and early opacification of the collateral vein (**D**, dashed white arrow), which communicate with the left common iliac vein (CIV) (**E**, white arrow). The collateral vein could be described as a duplication of the inferior vena cava or ascending lumbar vein rather than the left gonadal vein (LGV). **(F)** Venogram showed stenosis of the LRV with contrast attenuation over the abdominal aorta (black star) and large refluxing drainage pathway (white star). The LRV could be approached from the right internal jugular vein (via the inferior vena cava) (black arrow) and the left common femoral vein (FV) (via the drainage vein) (dashed black arrow). **(G)** After LRV stenting, the collateral pathway has disappeared and the LRV is well opacified. **(H)** Three-month postoperative photograph showing healing of the skin ulcers.

A 26-year-old man with a history of bilateral great saphenous vein stripping presented to our hospital with recurrent ulcers over the lateral ankle and forefoot bilaterally (Fig 3, A-C). DUS showed no superficial venous insufficiency and slight segmental reflux in bilateral FVs. CTA revealed LRV compression with dilatation and early opacification of the collateral vein communicating with the left CIV (Fig 3, D and E). A venogram revealed LRV compression-induced collateral pathway reflux draining directly into the left CIV (Fig 3, F). We performed LRV stenting (SMART 14 × 60 mm, Cordis Corporation) using the jugular approach.^3^^,^^4^ After stenting, the collaterals disappeared, and the ulcers gradually healed (Fig 3, G and H). The patient was on antiplatelet therapy for six months, and at the final follow-up 44 months after intervention, the stent was patent with no recurrence of symptoms.

In this case, the drainage pathway here could be described as a duplication of the inferior vena cava or ascending lumbar vein rather than the LGV. Regardless, it functioned as a collateral reflux flow, in which LRV compression-induced increased venous pressure was transmitted and compensated to a more caudal zone; therefore, the term "LGV" has been used.

## Discussion

The three cases had the following common findings: (1) the clinical manifestations and course were typical of DVI-LE; (2) LRV compression-induced LGV reflux drained into the iliac veins; (3) no iliac vein obstruction was observed; and (4) clinical improvement was observed after LRV stenting. Two of the three patients presented with bilateral DVI-LE symptoms.

**Fig 4:**
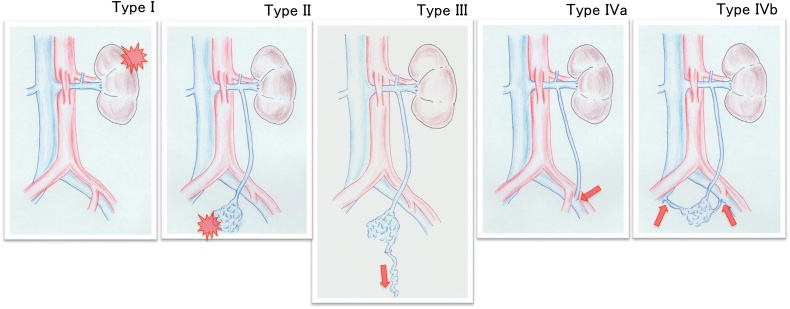
Various hemodynamics with left renal vein (LRV) compression and the collateral vein reflux. In type I, the collaterals do not compensate for LRV compression, presents with hematuria and/or flank pain. In type II, the gonadal venous reflux decompresses venous hypertension and drains into the pelvic plexus, presents with pelvic symptoms. In type III, there are extrapelvic varices via the pelvic escape point (PEP). In type IV, the PEP is located in the iliac veins with clinical presentations of deep venous insufficiency of the lower extremities, can further be classified as IVa, with direct communication with the iliac veins, and IVb, through the plexus.

Meissner et al^1^^,^^2^ reported the concept of PeVD and the LRV compression-induced increased venous pressure being transmitted to a more caudal venous reservoir by collateral reflux flow. The clinical presentation of PeVD vary according to the final venous reservoir site as determined by the drainage pattern and the pelvic escape point (PEP) and are classified as follows: (1) flank pain, hematuria, (2) chronic pelvic pain, (3) venous claudication (iliac venous obstruction), and (4) lower extremity varicosities.^1^ Our three cases were categorized as PeVD. However, some differences existed: (1) the PEP communicated with the iliac veins and (2) the presentation was that of DVI-LE, with possible involvement of both lower extremities. Based on current evidence, this study’s pathophysiology is shown in Fig 4. Type I, in which collaterals do not compensate for LRV compression, presents with hematuria and/or flank pain. Type II, in which LGV reflux decompresses venous hypertension and drains into the pelvic plexus, presents with pelvic symptoms. Type III results in extrapelvic varices via PEP. Type IV, in which the PEP is located in the iliac veins with clinical presentations of DVI-LE, can further be classified as IVa, with direct communication with the iliac veins (case 3), and IVb, through the plexus (cases 1 and 2). Even if the PEP in the iliac veins is located on either side, the clinical symptoms can occur in either or both lower extremities, suggesting a probable volume overload to the entire iliocaval region. This might be considered a “venous hypertension disease” or a similar spectrum.

Owing to the varying FV reflux degree among the three cases, it remains uncertain how the deep venous reflux contributed to this condition. However, it seemed to have a similar pathophysiology to PTS, which presents with an outflow obstruction with or without distal reflux. LRV compression with LGV reflux may present similar hemodynamics to PTS and NIVL, which cause DVI-LE.

CTA is useful in the diagnosis, evaluation of LRV compression, detection of LGV early opacification, and the presence or absence of iliac venous obstruction with a single examination.^3^^,^^4^ It also allows the exclusion of other arteriovenous fistula diseases, such as iatrogenic injury, neoplasms, and erosion of an arterial aneurysm that could demonstrate early opacification in the venous segment. DUS examination is also useful; however, LRV and LGV are not commonly evaluated when the clinical presentations are in the lower extremities. We did not measure the LRV compression-induced pressure gradient because venous blood pressure values are significant in a standing position. Furthermore, LRV compression with LGV reflux may be asymptomatic; thus, treatment indications should be based on clinical symptoms rather than the pressure gradient.^5^, ^6^, ^7^

Therefore, the patient selection criteria for treatment of this condition are (1) symptomatic DVI-LE, but PTS or NIVL is ruled out as the etiology, resulting in compression therapy as the only treatment option, (2) LRV stenosis with LGV reflux on imaging findings, and (3) the hemodynamics are type IV in Fig 4.

Because all our cases had LRV compression-induced secondary gonadal vein reflux, LRV stenting and not gonadal vein embolization was performed.^4^^,^^8^ All cases were treated successfully without complications. However, because a serious complication of stent migration has been reported,^3^^,^^4^^,^^8^, ^9^, ^10^ careful interventional techniques, large-diameter stents, and close follow-up with DUS are necessary, and patient selection should be limited to severe cases.

## Conclusions

LRV stenosis with GV reflux may cause DVI-LE and should be considered when a recalcitrant ulcer owing to DVI is suspected, but neither PTS nor NIVL can be ruled out. LRV compression caused secondary gonadal vein reflux resulting in volume overload in the iliac veins and DVI-LE in our three cases. Clinical improvement was observed with LRV stenting in all three patients.
